# Brain morphometry shows effects of long-term musical practice in middle-aged keyboard players

**DOI:** 10.3389/fpsyg.2013.00636

**Published:** 2013-09-23

**Authors:** H. Gärtner, M. Minnerop, P. Pieperhoff, A. Schleicher, K. Zilles, E. Altenmüller, K. Amunts

**Affiliations:** ^1^Institute of Neuroscience and Medicine (INM-1), Forschungszentrum JülichJülich, Germany; ^2^Department of Psychiatry, Psychotherapy, and Psychosomatics, RWTH Aachen UniversityAachen, Germany; ^3^Institute of Music Physiology and Musicians' Medicine, Hannover University of Music, Drama and MediaHannover, Germany; ^4^C. & O. Vogt Institute for Brain Research, Heinrich Heine University DüsseldorfDüsseldorf, Germany

**Keywords:** brain plasticity, long-term musical practice, musicians, MRI, deformation-based morphometry, DBM, cerebral cortex

## Abstract

To what extent does musical practice change the structure of the brain? In order to understand how long-lasting musical training changes brain structure, 20 male right-handed, middle-aged professional musicians and 19 matched controls were investigated. Among the musicians, 13 were pianists or organists with intensive practice regimes. The others were either music teachers at schools or string instrumentalists, who had studied the piano at least as a subsidiary subject, and practiced less intensively. The study was based on T1-weighted MR images, which were analyzed using deformation-based morphometry. Cytoarchitectonic probabilistic maps of cortical areas and subcortical nuclei as well as myeloarchitectonic maps of fiber tracts were used as regions of interest to compare volume differences in the brains of musicians and controls. In addition, maps of voxel-wise volume differences were computed and analyzed. Musicians showed a significantly better symmetric motor performance as well as a greater capability of controlling hand independence than controls. Structural MRI-data revealed significant volumetric differences between the brains of keyboard players, who practiced intensively and controls in right sensorimotor areas and the corticospinal tract as well as in the entorhinal cortex and the left superior parietal lobule. Moreover, they showed also larger volumes in a comparable set of regions than the less intensively practicing musicians. The structural changes in the sensory and motor systems correspond well to the behavioral results, and can be interpreted in terms of plasticity as a result of intensive motor training. Areas of the superior parietal lobule and the entorhinal cortex might be enlarged in musicians due to their special skills in sight-playing and memorizing of scores. In conclusion, intensive and specific musical training seems to have an impact on brain structure, not only during the sensitive period of childhood but throughout life.

## Introduction

“*Ce qu'on ne peut pas dire et ce qu'on ne peut pas taire, la musique l'exprime*^1^.” This well-known statement by Victor Hugo illustrates the deep impact of music on human beings. In the past scientists frequently investigated to what extent music affects individuals and whether it has measurable effects on the brain and/or physical health. A recent review outlined the effect of music on health through changes in different domains, e.g., reward, immunity or social affiliation, in the context of neurochemical systems like neurotransmitters, hormones and peptides (Chanda and Levitin, 2013). Moreover, the particular need for specific requirements for musicians such as motor training, auditory capacities, and memory have been supposed to allow conclusions to be made about the relation between music, brain plasticity and behavior (for a recent review, see Herholz and Zatorre, 2012).

For example, pianists showed left-hand superiority in motor tasks when compared to non-musician controls (Amunts et al., 1997; Jäncke et al., 1997). These behavioral differences were associated with changes in brain structure. Musicians had a reduced interhemispheric asymmetry of the intrasulcal length of the precentral gyrus, an estimate of the size of the motor cortex controlling hand movement; this measure was also larger in keyboard players than in controls (Amunts et al., 1997; Li et al., 2010). Others reported a different degree of lateralization depending on auditory habits and instrument choice, respectively (Schneider et al., 2005; Bangert and Schlaug, 2006). A correlation between gray matter density of the right and left sensorimotor cortices in controls but not in musicians was found, and interpreted as a correlate of the independent use of pianists' hands (Lv et al., 2008). Differences in the corpus callosum between musicians and non-musicians have been reported in the anterior midbody, which is thought to connect motor areas of both hemispheres (Schlaug et al., 1995; Lee et al., 2003). Musicians had a larger cerebellum than controls (Hutchinson et al., 2003), and they showed a larger primary auditory cortex (Bermudez et al., 2009) and planum temporale (Bermudez and Zatorre, 2005). The latter is involved in pitch processing and has been shown to be markedly lateralized to the left among musicians with absolute pitch (Schlaug et al., 1995; Keenan et al., 2001; Luders et al., 2004). Recently, a relation between auditory habits and brain function has been revealed: during listening to an unknown piece of music, a high reward value was associated with increased activation in the nucleus accumbens (Salimpoor et al., 2013).

Correlations between brain structure and age of onset or years of musical training supported the hypothesis that the observed differences in brain structure are directly influenced by the amount of practice. In this context it has been suggested that there might be a sensitive period during which the impact of training on brain structure is particularly high (Penhune et al., 2005). For example, an early age of onset of musical practice was associated with enlarged cortical representation of the fingers of the left hand in string players (Elbert et al., 1995), with longer intrasulcal length of the precentral gyrus as a measure of motor cortex size (Amunts et al., 1997), and with higher diffusivity measures along the corticospinal tract (Imfeld et al., 2009). Recently it was shown that early-trained musicians had greater “connectivity” as estimated by the fractional anisotropy (FA) in the posterior midbody and isthmus of the corpus callosum and that FA in this region was related to age of onset of training and sensorimotor synchronization performance. It was proposed that training before the age of 7 years results in changes in white-matter connectivity that may serve as a scaffold upon which ongoing experience can build (Steele et al., 2013).

To date only a few longitudinal studies have been conducted, analyzing long-lasting effects of musical training on brain structure. After 1 year of musical practice, children showed better fine motor and auditory discrimination skills than controls without practice (Schlaug et al., 2005). Further studies reported temporarily improved spatial abilities (Costa-Giomi, 1999), as well as gray matter changes in motor and auditory regions and the corpus callosum (Hyde et al., 2009a,b) after receiving piano lessons.

Apparently, many studies have found differences between musicians and non-musicians, which altogether strongly support the notion of plasticity in the human brain due to training. However, all studies mentioned above investigated children or young adults under the age of 30. There are only a few studies of musicians in higher age groups. Pascual-Leone et al. (1995) showed in older subjects that short-term training of a piano exercise over a period of 5 days led to improved motor performance and modulation of the cortical motor output to muscles involved in a motor task using transcranial magnetic stimulation. The subjects in this experiment had a mean age of 44, but only three participants per group were analyzed. Sluming et al. (2002) investigated Magnetic Resonance Imaging (MRI) data of male orchestra musicians (playing a variety of instruments) with an age range of 26–66 and found an increased gray matter density in the left inferior frontal gyrus (Broca's Area). Different abilities of orchestral musicians such as music discrimination, visuospatial and audiospatial localization, musical syntax processing and sight-reading were supposed to underlie this finding although the parameter “gray matter density” is difficult to interpret in biological terms. Additionally, higher age was associated with lower gray matter volume only in control subjects, leading to the supposition that the long-term musical training might reduce degeneration processes through the lifespan. Other data suggested that attention-dependent processing of a mistuned harmonic was enhanced in older musicians, and thus provided evidence that age-related decline in hearing abilities was mitigated by musical training (Zendel and Alain, 2013).

Considering the above mentioned data on volumetric changes in cortical regions and fiber tracts associated with sensorimotor processing in young musicians, we examined to what extent musical training does shape the structure of the middle-aged brain, depending on intensity of practice and qualification. It is yet unclear, whether changes in brain structure which have for example been observed in children after intensive musical training (Hyde et al., 2009a,b) are still detectable at a higher age. Therefore, brains of middle-aged, intensively practicing pianists and organists were investigated in the current cross-sectional study and compared with age-matched controls using Deformation-based morphometry. Regions of special interest were the motor cortices and other motor regions, the corpus callosum and the auditory cortices, as former studies revealed differences in younger subjects in these regions (Gaser and Schlaug, 2003; Bermudez et al., 2009).

A region-of-interest-based statistical analysis on 3 Tesla T1-weighted MRI data was employed to determine the extent of structural differences between the brains of two groups of musicians with different practice regimes and qualification and control subjects.

## Materials and methods

### Subjects

Twenty right-handed, male musicians (mean age 43.3 ± 3.8 years) and 19 matched controls (mean age 43.5 ± 3.8 years) were included in the study (Table 1). All of them participated after written informed consent. The study was approved by the local ethics committee. Nine musicians and 10 controls who had already participated in a previous MR study (Amunts et al., 1997) were enrolled again in the present study to obtain new MR scans and new behavioral data from them. The subjects came from all over Germany and Switzerland. Additional musicians were recruited from music conservatories, through personal contacts and online search. All 20 musicians had studied the piano at a conservatory at least as a subsidiary subject. After following different career paths until study participation, 13 musicians were presently intensively practicing keyboard players (8 accompanists, 3 organists, and 2 chamber musicians, referred to as “M1”). The remaining seven were either music teachers at schools or string instrumentalists (3 teachers, 2 violinists, 2 bassists, referred to as “M2”). Musicians of both groups (M1, M2) were matched for age of onset and amount of practicing years (Table 1). Some of the controls had played an instrument before, but not professionally, and either for less than 2 years or more than 20 years ago. Subjects did not suffer from neurological or psychiatric disorders. A dementia detection test (DemTect; Kalbe et al., 2004) was conducted to screen out pathological memory decline. Hand preference was assessed using a questionnaire according to the Edinburgh Handedness Inventory (Oldfield, 1971). In this inventory, a laterality quotient (LQ) was calculated from hand preference in everyday activities. Subjects with a quotient above 40 were defined as consistent right-handers.

**Table 1:** **Participants**.

	**Age (y)**	**Practice commencement (y)**	**Amount of practice years**	**Practicing time (h/day)**
M1 (*n* = 13)	43.3 ± 3.4	7.8 ± 2.6	36.2 ± 4.5	2.8 ± 0.9
M2 (*n* = 7)	43.3 ± 4.8	7.9 ± 1.8	35.4 ± 5.2	1.8 ± 0.9
C (*n* = 19)	43.5 ± 3.8	–	–	–

M1, intensively practicing musicians; M2, less intensively practicing musicians; C, controls.

### Questionnaire and behavioral tests

All subjects answered a musical biography questionnaire that had been tested and successfully applied in previous studies (Jabusch et al., 2008; Granert et al., 2011) and that was adopted for the current study. Twelve questions relating to the following topics were answered: onset of musical practice (piano, other instruments), main professional music genre (classic, pop, jazz), musician status (amateur, professional), subject of study and final degree, main professional activity (soloist, accompanist etc.), daily practicing time during different periods in life (piano, other instruments, 5 and 10 years each period, respectively), current neurologic diseases, pain in the context with performing music, current medication, impairment during daily fine motor skills, questions concerning hand preference. From the estimated daily practicing times, an overall lifetime average practicing time per day was calculated.

Three tests were performed to examine the hand motor performance of the participants. The complete behavioral testing took ~60 min for each participant. (1) In a finger tapping test the subject had to type the space-bar of a keyboard as fast as possible within 20 s, at first using the right and then the left index finger. Raw scores show the tapping speed for right and left hand. An index was calculated (R – L)/(R + L) with the value zero indicating a perfectly symmetric motor performance. (2) The Hand Dominance Test (HDT) included three different tasks (tracing lines, dotting circles, dotting quadrates), each performed with the right and left hand separately (Steingrüber, 1971). An index above zero denoted right-handedness in each subtest and sums of sub scores were calculated. (3) The third hand motor test was the Contralateral Co-Movement-test (CoMo). It was originally developed to investigate involuntary co-movements of the contralateral hand or foot (not performing afore trained motor tasks) between patients with amyotrophic lateral sclerosis and controls, pointing toward corpus callosum dysfunction (Bartels et al., 2008). Being placed behind a visual barrier the subjects were unable to see their hands. Their feet were similarly out of sight, being placed under the table. The subjects were asked to carry out various motor tasks, such as making a fist, turning their hand over or spreading their toes successively with their right hand, left foot, left hand and right foot. The movements included in the official version of the test (Bartels et al., 2008) were extended to include two musician-specific motions (solely tapping of the ring finger; alternately tapping of thumb and little finger; each as fast as possible). Each involuntary contralateral co-movement was noted by the observer (complete co-movement = 2 points, partial co-movement = 1 point, no co-movement = 0 points). An individual score was calculated as a percentage of the maximum score (68 points). In musicians, fewer involuntary co-movements with the contralateral hand (not performing the task) were expected while performing the CoMo tasks because of the well-trained independence of their hands. No significant difference had been expected for the lower extremity so these results were not included in the statistical analysis. Tapping test and HDT were analyzed using a One-Way ANOVA in SPSS software for Windows (version 20) to test between-group differences (*p* < 0.05). Due to the skewed data for the statistical analysis of the CoMo a combination of Kruskal Wallis test with Monte Carlo permutation was used to compare the distributions of the three groups.

### MR imaging

Structural magnetic resonance images were acquired with a Siemens Magnetom Trio 3 Tesla scanner and a 12 channel head coil (Siemens, Erlangen, Germany). A 3D T1-weighted magnetization prepared rapid gradient echo (MP-RAGE) sequence with the following parameters was accomplished: 176 sagittal slices, voxel size 1 × 1 × 1 mm, repetition time 2250 ms, echo time 3.03 ms, inversion time 900 ms, field of view 256 × 256 mm, flip angle 9°, acquisition matrix 256 × 256. All images were visually inspected to prove image quality and to rule out brain lesions.

MR images were segmented using tools in SPM8 (Ashburner and Friston, 2005; http://www.fil.ion.ucl.ac.uk/spm/software/spm8/), yielding masks of gray and white matter. These were combined to a binary mask of the brain. The contour of each mask was superimposed onto the MR image, in order to visually inspect and manually correct it, if necessary. MR images of the brains were corrected for inhomogeneity by means of the software package N3 (Sled et al., 1998) and registered to the T1-weighted single subject template of the Montreal Neurological Institute (“colin27”; Holmes et al., 1998; Evans et al., 2012). The program FLIRT from the FMRIB software library (Jenkinson et al., 2002; http://fsl.fmrib.ox.ac.uk/fsl/fslwiki/FLIRT) was used for affine registration. After that the MR images were non-linearly registered with a program that models an elastic deformation (Hömke, 2006). The deformation was driven by forces which are calculated based on the squared difference in voxel-wise intensity differences between the reference image and the transformed image. The resulting transformation of each brain is defined by a deformation field, which is analyzed in the next step.

### Deformation-based morphometry (DBM)

The deformation fields were used to compute maps of voxel-wise volume differences of each individual brain relative to the reference brain [= Local Volume Ratio (LVR)-maps; (Pieperhoff et al., 2008)]. The LVR is the exact volume difference of each finite volume element (i.e., voxel) relative to the reference brain, that is determined by a given deformation field. It differs from the Jacobian determinant, which is often used for the same purpose, in that the latter requires the computation of partial derivatives of the deformation field. These, however, can only be approximated on discrete grids. Depending on the approximation scheme, the maps of volume differences calculated by the Jacobian determinant can be less smooth than the LVR maps.

The LVR maps were analyzed in two ways: to begin with, volumes of anatomical regions were calculated, which were defined by cytoarchitectonic maps of cortical areas and subcortical nuclei (http://www.fz-juelich.de/inm/inm-1/EN/Forschung/_docs/Gehirnkarten/gehirnkarten_node.html; Amunts et al., 2007; Zilles and Amunts, 2010), myeloarchitectonic maps of fiber tracts (Bürgel et al., 2006), maps of gyri and basal ganglia from the ICBM-atlas of the colin27 reference brain (http://www.loni.ucla.edu/ICBM/Downloads/Downloads_ICBMtemplate.shtml), as well as segmentations of hemispheres, cerebellum, corpus callosum and ventricles, which were done by ourselves. Thus, 319 regions were examined. Subsequently, voxel-wise volume differences between all groups were computed. The LVR-maps were spatially smoothed for this step, using an isotropic Gaussian kernel (full width at half maximum = 2 mm). The intracranial space was manually delineated in the sagittal sections of each subjects' MR image in order to measure the intracranial volume (ICV), which was used to correct for head size differences (Mathalon et al., 1993).

In the first step of the statistical analysis, a principle component analysis (PCA) was calculated in order to reduce the large amount of region data to a limited number of components which explained most of the structural variability. Only volumes of cytoarchitectonic and myeloarchitectonic regions, as well as of basal ganglia and brain stem structures were evaluated, i.e., structures which are microstructurally and functionally defined entities of the brain (Amunts et al., 2007). The PCA was separately calculated for regions of the left and right hemisphere. The subjects' scores of the first eight components (which explained about 70% of the structural variability) were tested by a multivariate analysis of variance (MANOVA) for differences between groups. In the next step, volumes of anatomical regions were statistically analyzed for differences between the subject groups by means of a One-Way analysis of covariance (ANCOVA) with the ICV of each subject as covariate. For these computations, SAS 9.3 (SAS-Institute, Cary, NC) was applied. First of all, an *F*-Test was performed in each of the 319 regions to look for overall differences. After that, pairwise comparisons were calculated for the three contrasts (M1 vs. C, M1 vs. M2, and M2 vs. C). The results of those regions are reported in Table 3, where the *t*-score of the group differences corresponds to a *p*-value below 0.05 (uncorrected for multiple comparisons). In addition, a voxel-wise analysis of the same ANCOVA model was pursued in order to visualize the results from the ROI-analysis. Particularly, the voxel-wise results can display in which sub-area of a predefined ROI the differences occur. SPM8 was applied for the voxel-wise analysis, which yielded a map of *t*-scores for each pairwise group comparison. These maps were superimposed to the MNI-single subject reference brain. In Figures 4–6 the voxel-wise results are presented at a threshold of *t* = 2.03 (corresponding to *p* < 0.05).

## Results

### Motor and behavioral data

All participants were consistent right-handers according to the Edinburgh Handedness Inventory. They reached a LQ of at least 50 with an average of 81.42 (right-handedness: above 40) except one musician (organist, *LQ* = 0, thus ambidextrous).

**Figure 1:**
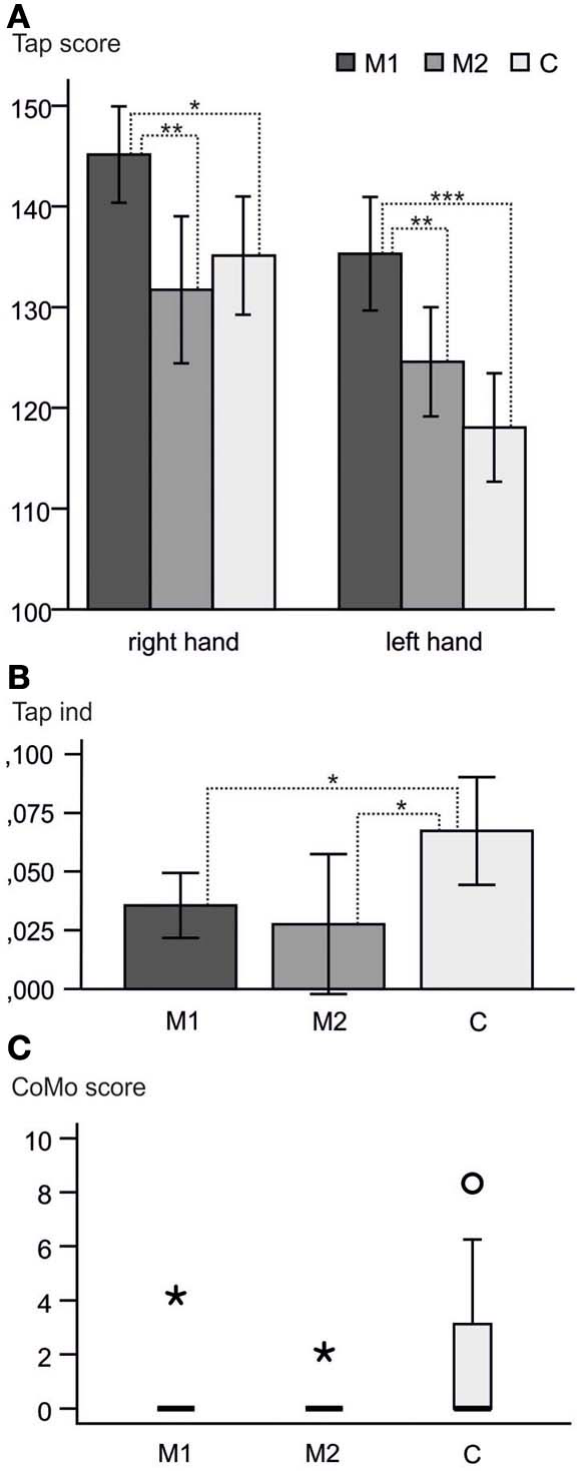
**Behavioral results. (A)** Raw tapping scores right and left hand and 95% confidence intervals. M1 reached higher scores than M2 and C in both hands. No significant difference was found between M2 and C (*p* > 0.05). **(B)** Means and 95% confidence intervals of the tapping indices. M1 and M2 showed significantly lower tapping index means than C, indicating a more symmetric motor performance. **(C)** Boxplot of the CoMo scores. Only two of the musicians showed co-movements at all (one in each of the subgroups; asterisks). Round circle = outlier within the control group. ^*^p < 0.05; ^**^p < 0.01; ^***^p < 0.001.

Hand performance revealed differences not only between musicians and controls, but also within the musicians, i.e., between M1 and M2 subgroups: M1 musicians showed the highest tapping scores (Figure 1A, being significantly faster with both hands than controls and M2 musicians). No significant difference in tapping speed was found between M2 musicians and controls, although there was a tendency in the left hand of M2 musicians to be faster than controls.

The tapping index differed significantly between the three groups (One-Way ANOVA with tapping index as a dependent variable; *p* = 0.026, η^2^ = 0.183). This overall difference was due to the difference between musicians and controls without an effect between the two musician groups. M2 were even more symmetric than M1, but showed greater variability in the indices (Figure 1B).

In the HDT all participants showed an average score of 30.04 ± 13.66 (right-handedness > 10). No difference between musicians and controls was found in the HDT concerning the degree of right-handedness (*p* = 0.644, η^2^ = 0.024).

A significant difference between musicians (M1 + M2) and controls was found for the upper extremity (*p* = 0.016) in the CoMo. As expected the musicians were better able to control the movement of one hand without accidentally moving the contralateral hand as well. Only two of the musicians showed co-movements at all (one in each of the subgroups). There was no significant difference between M1 and M2 (Figure 1C).

**Figure 2:**
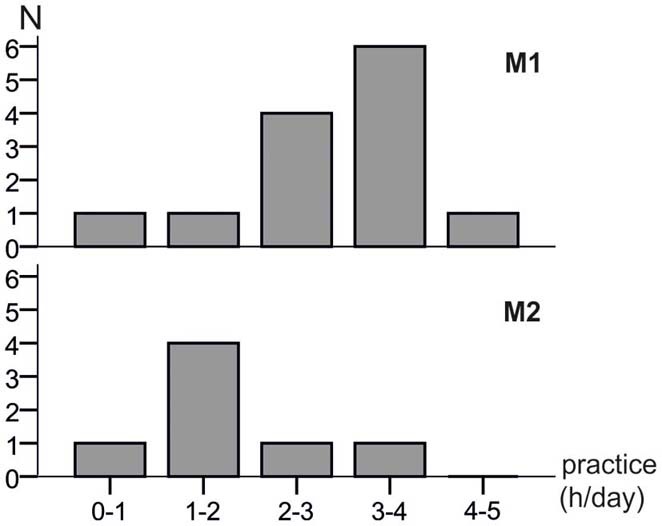
**Distributions of the estimated daily practice times of both musician groups.** M1 musicians had a significantly longer practicing time per day (*t*-test of the means: *p* = 0.044).

The distributions of the estimated average daily practicing times are shown in Figure 2 with M1 practicing significantly more than M2 (*t*-test of the means: *p* = 0.044). Whereas eleven M1 musicians practiced more than 2 h per day and only two practiced less, two of the M2 musicians practiced more than 2 h per day while the remaining five practiced less.

### Brain morphometry

#### Principal component analysis (PCA)

The eigenvalues and corresponding proportions of variance, which were explained by each component, are listed in Table 2. The eigenvalues of corresponding components were nearly equal between both hemispheres. The first eight components summarized about 69 and 70% of variance in left and right hemisphere, respectively. The major constituents of the first component were subcortical structures like thalamic nuclei, basal ganglia, substantia nigra, red nucleus, periaquaeductal gray matter, cholinergic nuclei as well as amygdala and the cortico-spinal tract. Areas of motor and somatosensory cortex, inferior and superior parietal lobule had a major contribution to the second component in both hemispheres, but areas of the auditory cortex as well as the corticospinal tract predominantly in the right hemisphere. The anatomical contributions to the subsequent components were less distinctively associated among each other.

**Table 2:** **PCA results**.

**Comp**	**Eigenval**	**Prop**	**Cum**
**A. LEFT HEMISPHERE**			
1	33.33	0.267	0.267
2	11.27	0.090	0.357
3	10.47	0.084	0.441
4	7.95	0.064	0.504
5	6.99	0.056	0.560
6	5.97	0.048	0.608
7	5.12	0.041	0.649
8	4.61	0.037	0.686
**B. RIGHT HEMISPHERE**			
1	32.12	0.257	0.257
2	13.45	0.108	0.365
3	11.24	0.090	0.455
4	7.77	0.062	0.517
5	6.94	0.056	0.572
6	6.18	0.049	0.622
7	4.86	0.039	0.661
8	4.47	0.036	0.696

Eigenvalue and corresponding proportion of variance assigned to each principal component of the region volume data (range of proportion, cumulative 0–1). Abbreviations: Comp, component; Eigenval, eigenvalue; Prop, proportion; Cum, cumulative.

**Figure 3:**
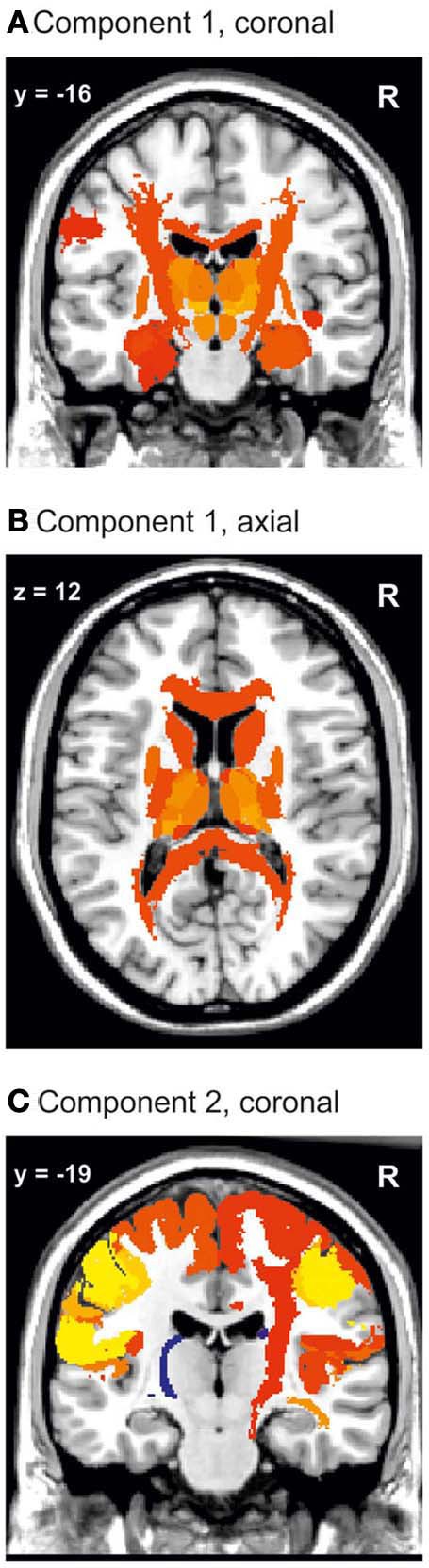
**Visualization of the first and second PCA component.** Component 1 **(A,B)** includes the whole corpus callosum and the thalamic nuclei. In **(C)** it can be seen that the major constituents of component 2 are the sensorimotor cortices and the right corticospinal tract. Color coding: red to yellow, coefficient of the eigenvalue > 0.1. Dark blue to light blue, coefficient of the eigenvalue < −0.1.

The MANOVA of the first eight principle components revealed differences in brain structure between the three groups [tested by Wilks' Lambda: *p* = 0.0167 (right hemisphere) and *p* = 0.0468 (left hemisphere)]. This result justified to perform univariate tests for differences between groups in each variable, i.e., component (Rencher and Scott, 1990; Rencher, 2002). The second component showed differences between the groups. The regions which had major contributions to the first two components are visualized in Figure 3.

#### Univariate analysis of group differences

**Figure 4:**
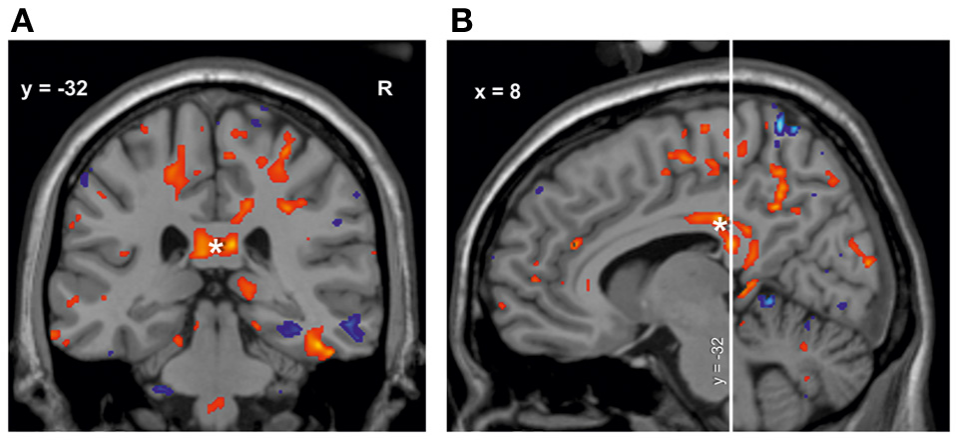
Volume change of M1 musicians compared to controls in the corpus callosum^*^. (A) Coronal slice (*y* = −32). **(B)** Sagittal slice (*x* = 8). The significant cluster is located in the isthmus and splenium of the corpus callosum, with few voxels reaching the posterior midbody.

**Figure 5:**
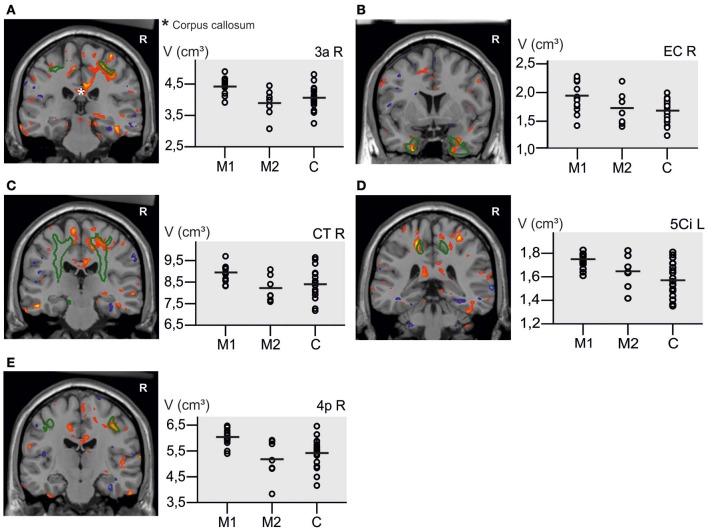
**Results from voxel-based and ROI-Analysis are overlaid.** Volume increase (red) and volume decrease (blue) in M1 musicians compared to controls from the voxel-based analysis are used for visualization of the ROI results in five coronal slices (height threshold *t* = 2.03, threshold corresponds with *p* < 0.05, uncorrected for multiple comparisons). As green curves the contours of the regions used in the ROI-analysis are outlined. The scatterplots display the absolute volume (in cm^3^) within the three groups in the corresponding regions. Horizontal lines = volume means. Contours: **(A)** area 3a (somatosensory cortex), **(B)** entorhinal cortex (EC), **(C)** corticospinal tract (CT), **(D)** left area 5Ci (superior parietal lobule), **(E)** area 4p (primary motor cortex). Red, volume increase; blue, volume decrease.

**Figure 6:**
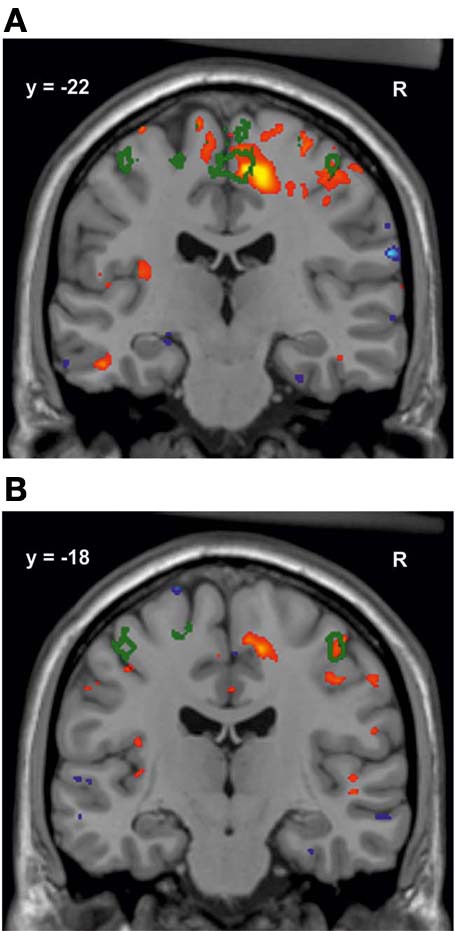
**Volume increase in M1 musicians compared to M2 musicians in the right primary motor cortex (red) with the contour of area 4a (green). (A)** Shows a volume increase mainly in the foot region, but also the hand region (Geyer et al., 1996; **B**).

In the subsequent univariate analyses of single brain regions, M1 musicians showed significantly greater volumes in several regions as compared to M2 musicians (Table 3A) and controls (Table 3B). Only results from those regions are reported in which the preceding *F*-test had reached significance (*p* < 0.05). Volumetric differences between M1 and M2 musicians were most pronounced in the right somatosensory (area 3a, 3b, 1, 2; Geyer et al., 1999, 2000; Grefkes et al., 2001), and motor (area 4a, 4p, 6; Geyer et al., 1996; Geyer, 2003) cortex and in the right corticospinal tract and cingulate bundle (Bürgel et al., 2006). However, additional differences were found with respect to the left corticospinal tract, left area 4 and the left red nucleus with M1 musicians having larger volumes than M2 (Table 3A). A similar set of regions was larger in M1 musicians as compared to controls (Table 3B). Additionally, effects between M1 and controls were seen in the left superior parietal lobule (area 5Ci; Scheperjans et al., 2008a,b), the posterior half of the corpus callosum (from *y* = −14 to *y* = −44, see Figure 4), and bilaterally in the entorhinal cortex and the thalamus. The localization of exemplary regions of interest is shown in Figures 5, 6.

**Table 3:** **Volume difference between the groups from the ROI-analysis**.

**Macroscopic localization**	**Area**	**Side**	**Map**	**Est**	**StdErr**	**Tmax**	***p*-value**
**A. VOLUME M1 > M2**							
Parietal cortex	Area 3b	R	1	1029	244	4.22	0.0002^***^
	Area 1	R	1	772	199	3.88	0.0004^***^
	Area 3a	R	1	478	133	3.60	0.0010^**^
	Postcentral gyrus	R	3	1855	596	3.11	0.0037^**^
	Area 2	R	1	628	221	2.85	0.0073^**^
Frontal cortex	Area 4p	R	1	715	197	3.62	0.0009^***^
	Area 4a	L	1	702	201	3.48	0.0013^**^
	Area 4a	R	1	515	192	2.69	0.0109^*^
	Area 6	L	1	1202	493	2.44	0.0199^*^
	Area 6	R	1	1020	451	2.26	0.0300^*^
	Area 4p	L	1	330	154	2.14	0.0395^*^
White matter	Corticospinal tract	R	2	562	162	3.46	0.0014^**^
	Corticospinal tract	L	2	427	174	2.46	0.0192^*^
	Corpus callosum (posterior)	−	3	933	435	2.15	0.0389^*^
Others	Cingulate gyrus	R	3	1718	590	2.91	0.0062^**^
	Red nucleus	L	3	24	12	2.06	0.0470^*^
**B. VOLUME M1 > C**							
Parietal cortex	Area 5Ci (SPL)	L	1	124	33	3.71	0.0007^***^
	Area 3a	R	1	314	102	3.08	0.0040^**^
	Area 3b	R	1	497	188	2.65	0.0120^*^
	Area 1	R	1	327	153	2.13	0.0402^*^
Frontal cortex	Area 4p	R	1	469	152	3.09	0.0039^**^
	Precentral gyrus	R	3	764	356	2.15	0.0387^*^
White matter	Corticospinal tract	R	2	323	125	2.59	0.0140^*^
	Cingulate bundle	R	2	96	38	2.56	0.0151^*^
	Corpus callosum (posterior)	–	3	847	334	2.54	0.0157^*^
Others	Entorhinal cortex	R	3	233	88	2.66	0.0118^*^
	Lateral geniculate body	R	3	5	2	2.32	0.0262^*^
	Thalamus IPU	R	1	11	5	2.32	0.0263^*^
	Entorhinal cortex	L	3	98	44	2.22	0.0333^*^
	Thalamus PO	R	1	9	4	2.07	0.0454^*^
**C. Volume C > M1**							
Parietal cortex	Area PFm (IPL)	R	1	406	178	2.28	0.0288^*^
	Area PGa (IPL)	R	1	351	171	2.05	0.0477^*^
	Inferior parietal lobule	R	3	613	299	2.05	0.0477^*^
**D. Volume C > M2**							
Parietal cortex	Inferior parietal lobule	R	1	1901	749	2.54	0.0158^*^
	Area 7p (SPL)	L	1	353	145	2.43	0.0204^*^
	Area 1	R	1	446	186	2.39	0.0224^*^
	Area 3b	R	1	532	228	2.33	0.0256^*^
	Area PGa (IPL)	R	1	477	208	2.29	0.0280^*^
	Precuneus	L	3	711	316	2.25	0.0307^*^
Frontal cortex	Area 4a	L	1	391	189	2.07	0.0455^*^

p-values (< 0.05) are given from an ANCOVA (with intracranial volume as covariate). Abbreviations: M1, M2, musician subgroups; C, controls; Map, definition of the region (1, cytoarchitectonic; 2, myeloarchitectonic; 3, macroscopic); Est, estimated volume change (mm^2^); StdErr, standard error; Tmax, maximal T-value; R, right; L, left; a, anterior/rostral; p, posterior/caudal; l, lateral; SPL, superior parietal lobule; 5Ci, area 5 near the cingulate sulcus; IPU, inferior pulvinar; PO, posterior nucleus; OFC4l, lateral orbitofrontal cortex; IPL, inferior parietal lobule; PFm, caudal part of the IPL on the supramarginal gyrus, transition zone between area PF and PG; PG, part of the IPL on the angular gyrus (PGa, rostral; PGp, caudal).

References to cytoarchitectonic maps: area 1, 3a, 3b, 4a, 4p, 6 (Geyer et al., 1996, 1999, 2000; Geyer, 2003); area 2 (Grefkes et al., 2001); area 5Ci, 7a, 7p (Scheperjans et al., 2008a,b); area PFm, PGa, PGp (Caspers et al., 2006, 2008). Fiber tracts: Bürgel et al. (2006).

* p < 0.05;

** p < 0.01;

*** p < 0.001.

The analysis also revealed regions, where controls had greater volumes than M1 and M2 musicians. Controls showed increased volumes as compared to M1 in the inferior parietal lobule (area PFm, PGa, PGp; Caspers et al., 2006, 2008) (Table 3C). Larger volumes than in M2 musicians were found in the inferior parietal lobule, the precuneus and the frontal cortex including area 4a (Table 3D). The effects in these regions were weaker (all *p*-values < 0.05, but above 0.01, see Tables 3C,D) than those mentioned above (Tables 3A,B). Results with *p* < 0.05 are listed in Table 3.

### Brain structure and behavior

**Figure 7:**
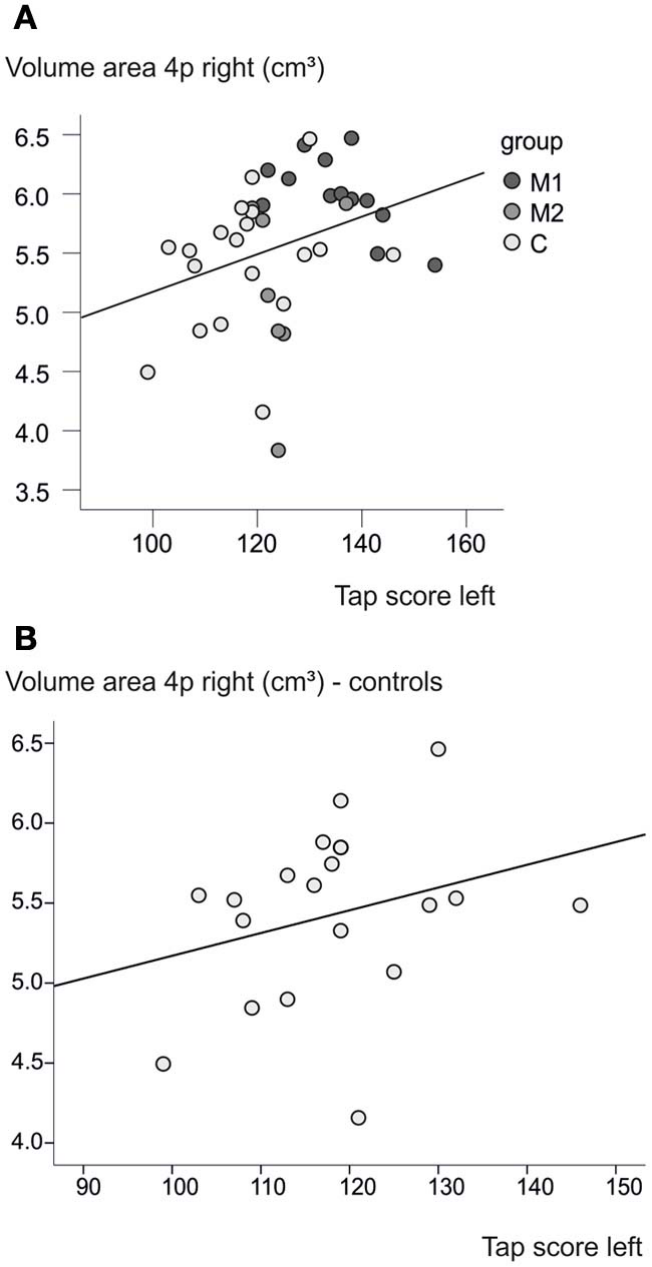
**Brain volume and behavior. (A)** A higher tapping score of the left hand is associated with a greater volume in right area 4p (*p* = 0.045, *R*^2^ = 0.102). **(B)** A slight effect can be seen also within the control group without the correlation analysis reaching significance (*p* = 0.23, *R*^2^ = 0.084).

A weak correlation between the tapping score of the left hand and the volume of the right motor cortex area 4p was found (*p* = 0.045, *R*^2^ = 0.102), when all subjects were taken into account. However, as can be seen in Figure 7, both variables in this correlation analysis are highly imbalanced between groups, so that this correlation may be influenced by the group differences, which were found for these variables independently from each other. An additional correlation analysis for each of the groups separately did not reach significance.

## Discussion

Studies in brains of musicians have contributed to our understanding of brain plasticity. It has been assumed that intensive experience in the motor or auditory domains conduces to long-lasting changes in the brain (Stewart, 2008; Kraus and Chandrasekaran, 2010; Herholz and Zatorre, 2012). It is less well-understood, however, in how far these effects can be detected not only in young musicians, but throughout the lifespan, and whether they may disappear in older ages, if musical practice becomes less intensive than during the period of education. More or less intensive musical practice is a factor contributing to intersubject variability among musicians, which has to our knowledge not yet been addressed. The current study has analyzed musicians with less or more intensive musical practice regimes, which was quantified through a musical biography questionnaire (Jabusch et al., 2008; Granert et al., 2011). In contrast to most previous studies, which have focused on young adults, the present investigation has analyzed middle-aged participants. All musicians had studied the piano at conservatories, at least as a subsidiary subject; i.e., they were all professional musicians. However, later they followed different professional careers, which allowed subdividing them into two subgroups: on the one hand keyboard players with intensive practice regimes and on the other hand music teachers and string instrumentalists, who practiced less intensively.

### Motor function and hand independence

Not surprisingly, musicians showed a superior motor performance of both the left and the right hand. This was demonstrated by higher tapping scores in M1 musicians, whereas M2 musicians were more similar to controls. In addition, musicians had lower tapping indices, indicating a better symmetric performance than controls. This difference can be explained by the intensive bimanual (for keyboard players) and left-hand (for string instrumentalists) training, respectively. A reduced degree of hand skill asymmetry in the same tapping test in keyboard players has already been shown previously (Jäncke et al., 1997). The lower tapping index of M1 musicians compared to controls was mainly due to their higher tapping scores of the left hand. Compared to M2 musicians, the tapping speed advantage of M1 musicians was stronger for the right hand. This seems to be evident, considering that M2 musicians consisted in part of string instrumentalists whose left-hand fingers are especially trained, whereas all M1 musicians were keyboard players. This result is consistent with a study of Elbert et al. (1995), which found a greater cortical representation of the left-hand fingers in string players using MR imaging and sensorimotor stimulation.

In contrast, the hand dominance test was not different between musicians and controls, although a previous study reported lower HDT scores in musicians than controls (Schlaug et al., 1995). We may speculate that compared to the tapping test (which proves the speed of both index finger taps as a task similar to requirements needed during piano practice), the HDT investigates more complex abilities, which are not as specifically needed in piano performance, and which are more similar to writing movements. This makes the test less sensitive to characterize hand movement in musicians as compared to controls.

Musicians showed significantly fewer co-movements than controls as tested in the CoMo. This is a clinical test, that had been developed to compare patients with amyotrophic lateral sclerosis and healthy subjects (Bartels et al., 2008). A significantly higher amount of contralateral co-movements as compared to healthy controls was associated with lower FA values in motor areas of the corpus callosum (Bartels et al., 2008). The current study revealed a greater capability to control hand independence in musicians than in controls. Such a capability may result from an improved transmission through the corpus callosum. In fact, larger regional volumes in the corpus callosum were found in musicians as compared to controls (see also below). It is also in line with studies investigating intracortical inhibition with transcranial magnetic stimulation (Ridding et al., 2000; Rosenkranz et al., 2007).

### Sensorimotor integration

The study demonstrated significant volumetric differences between musicians and controls in the primary and premotor cortex, the corticospinal tract, and the primary somatosensory cortex. These differences predominantly occurred in the right hemisphere, where the left hand is represented. Due to the bimanual training, it is not surprising that pianists have a better left-handed motor performance compared to right-handed, non-motoric-trained controls, which is in turn associated with structural changes of the corresponding right hemisphere. Differences in the primary motor cortex involved both hand and foot region. Playing on the pedal keyboard means a permanent motoric training of the feet for an organist. This is true, although to a lesser degree, for pianists, since control of the two pedals is a highly complex task involving subtle flexion-extension movements. More precisely, the pedaling in pianists is a highly refined skill requiring years of practice. Temporo-spatial control in the range of millimeters and milliseconds is required to modulate adaptively color, expressivity and loudness of the music. This could be the reason for the volume increase in the foot region in M1 musicians, including both pianists and organists. A lateralization of motor regions to the right hemisphere of musicians has also been revealed in diffusion tensor imaging (DTI) studies. For example, higher FA in piano players appeared in the right internal capsule (Bengtsson et al., 2005; Han et al., 2009). In this context it has to be mentioned that the results of several DTI analyses, which found differences in white matter between musicians and non-musicians, were contradictory. Namely, lower (instead of higher) FA values in musicians were shown in the internal capsule (Schmithorst and Wilke, 2002) and in the corticospinal tract (Imfeld et al., 2009). Additionally, the meaning of the different diffusion parameters is merely adequately understood, so that the authors have to be careful with the interpretation of their data (Jones et al., 2012) and tractography methods have to be further validated with postmortem data (Dell'Acqua and Catani, 2012).

In Figure 5 it can be seen that some of the controls had volumes comparable to those of the M1 musicians in the reported regions. Despite this overlap, the means in the M1 musician group are still higher than those of M2 musicians and controls. Furthermore, the lower variability within the M1 group compared to the control group could be an indicator for the specific musical training having converged the musicians' region volumes. In a range of different motor cortex sizes the M1 group consisting of highly specialized musicians might represent the superior part.

M1 musicians also showed significantly greater volumes than M2 in a very similar set of regions as mentioned for the comparison with controls. This difference between M1 and M2 musicians seems to reflect the difference in musical practice between the two groups. M1 musicians were mostly accompanists at music conservatories or organists, practicing during life course much more than M2 musicians. M2 musicians had studied the piano at least as a subsidiary subject during their studies but then either became music teachers at schools or professional string instrumentalists. Thus, they are presently not as specifically trained as M1 musicians. Furthermore, the volumetric differences between M1 and M2 musicians in anterior and posterior parietal, motor and premotor areas as well as subcortical regions correspond to the observed differences between these groups in hand performance scores and indices.

That even short training periods may lead to significant group differences between musicians and non-musicians has been shown in the past in two functional MRI (fMRI) studies (Karni et al., 1995; Pau et al., 2013). The present data therefore support the hypothesis that the specialization within the musicians had an influence on brain structure. Such a “specialization of the specialized” has already been pointed out by Bangert and Schlaug (2006). They discovered that the so-called omega sign in the precentral gyrus, which indicates the region of hand movement representation (Yousry et al., 1997), is oppositely lateralized in string instrumentalists and piano players (Bangert and Schlaug, 2006).

Moreover, no significant differences in motor regions were found between M2 musicians and controls. The markedly lower practicing time of M2 musicians may provide a plausible explanation. In other words, if there is a causal relation between motor training and measurable changes in human brain structure, this training has to be very specific and intensive.

Significant differences between M1 and both M2 and controls appeared not only in the premotor and primary motor cortex, but also in the somatosensory areas 3a, 3b, 1 and 2. During the performance piano players permanently modify the position and tension of the finger and hand muscles and the speed of contraction sequences while moving the keys, depending on the sensory information they receive from their fingertips. Sensory information available at finger-key contact in piano players seems to enhance the timing accuracy of finger movements, showing the importance of the tactile feedback (Goebl and Palmer, 2008). It is thus possible that tactile stimulation during long-term piano practice leads to structural alterations in the somatosensory regions of the brain.

The region of interest analysis also revealed extended volumetric differences in the posterior part of the corpus callosum between M1 musicians and controls (and to a smaller degree between both musician groups). This is in line with two studies showing changes in the anterior midbody of the corpus callosum between musicians and controls; this was interpreted as an indicator of greater interhemispheric connectivity between motor areas (Schlaug et al., 1995; Lee et al., 2003). Both studies, however, interpreted their findings on the basis of the Witelson classification, which was originally developed from rhesus monkey data (Witelson, 1989). Meanwhile, a new classification of the corpus callosum has been proposed, which differs in some aspects from the Witelson scheme (Hofer and Frahm, 2006). Based on human diffusion tensor imaging and fiber tractography data, it was shown that the fibers which connect the motor cortices run through the posterior instead of the anterior midbody of the corpus callosum. This scheme was later confirmed by another DTI and fMRI study (Wahl et al., 2007). The callosal changes in the current study mainly appeared in the isthmus and splenium, with a part of the cluster extending to the motor-fiber-carrying posterior midbody. According to Hofer and Frahm (2006), the isthmus carries fibers connecting both somatosensory cortices and the splenium carries those that connect the parietal, temporal and occipital lobes of both hemispheres. This also includes fibers between the auditory cortices. Differences between musicians and non-musicians in the posterior part of the corpus callosum had also been shown by Öztürk et al. (2002). They supposed that especially a continuous auditory training could lead to structural plasticity in this region. Later, a volume increase in the posterior part was shown in children after piano practice (Hyde et al., 2009a,b), which is also consistent with the results presented here. Finally, the changes in the corpus callosum corroborate the above mentioned hypothesis that fewer co-movements in the CoMo imply the musicians' improved callosal function with capability to control hand movements independently.

### Visuo-spatial coordination and memorizing skills

Further volumetric differences between M1 musicians and controls were shown in the left superior parietal lobule (area 5Ci). The SPL has been reported to play an important role for visuo-spatial capabilities and attention (Sergent, 1993; Hetland, 2000; Vaina et al., 2001; Gaser and Schlaug, 2003; Stewart et al., 2003), as well as for sensorimotor integration (Wolpert et al., 1998). These functions are highly relevant in musicians, e.g., sight-reading (Sergent, 1993). Sight-reading is defined as a complex process in which a musician reads and performs a score for the first time without having seen it before. Pianists, for example, have to read the score (often several notation systems at a time), press the right key (not to mention rhythm, musical interpretation, etc.) and anticipate the next passage while fluently playing what they have just read. Parallel to this, they have to integrate the visual information from the score to the corresponding spatial position on the keyboard. All this has to be done in a very short time as piano players reach frequencies of over 30 notes per second, as for example in the 6th Paganini-Etude by Franz Liszt (Münte et al., 2002). These specific requirements led to the term “sight-playing,” which is preferred among musicians because it underlines the importance of motor control abilities during sight-playing much better than the term “sight-reading” (Udtaisuk, 2005). Therefore, it is plausible that sight-playing might lead to a measurable volume increase in the superior parietal lobule if the training is intensive.

There was no significant difference in area 5Ci between either M1 and M2 musicians or M2 musicians and controls. However, M2 musicians showed a tendency toward a greater mean volume in left area 5Ci than controls and a smaller volume than M1 musicians. This finding suggested two aspects: first, M2 musicians might have been less trained in sight-playing abilities than M1-musicians, but of course better trained than controls. Second, an overall larger volume of 5Ci during education might have persisted for many years (at least in some of the M2 musicians), which could explain a higher volume compared to controls.

Another special requirement for the M1 musicians is the necessity to memorize scores. Accompanists have to play many different and challenging pieces during their daily work. Within musicians it is generally accepted that the time needed for practicing and preparing a piece for concerts gets shorter during the professional career. The subjects in the current study were on average 43 years old and can therefore be classed as highly experienced. When an accompanist plays a piece multiple times, it is advantageous for him to memorize fingerings as well as melodic and harmonic sequences from former performances. The entorhinal cortex has been reported to play a role for memory tasks and spatial navigation (Fyhn et al., 2004; Suthana et al., 2012). It is closely connected to the hippocampus which is one of the only two regions in the human brain (besides the olfactory bulb) in which neurogenesis has been reported (Eriksson et al., 1998). Entorhinal cortex and hippocampus are inter-connected, which shows the important role of the entorhinal cortex in memory processing (Lavenex and Banta Lavenex, 2013). Several studies have revealed training-induced changes of hippocampal structures, as for example in taxi drivers, who have to memorize a vast amount of information (Maguire et al., 2000). Another study showed greater hippocampal activation in musicians than controls during memory tasks as well as higher gray matter density (Groussard et al., 2010). Thus, it seems very plausible that there might be a relation between the larger entorhinal volume and the highly trained memorizing skills of the keyboard players.

These observed changes in brain structure may have already existed in childhood (either inborn, facilitating a successful career as musician, and/or as a consequence of early training) and perhaps remained constant during the subsequent years. An earlier study of our own group has found a correlation between the onset of musical training, and the size of the motor cortex as indicated by the depth of the central sulcus (Amunts et al., 1997). This correlation provides an argument, that the human motor cortex can exhibit functionally induced long-lasting structural adaptations, probably at the background of a specific genetic predisposition (Theusch and Gitschier, 2011; Morley et al., 2012; Park et al., 2012; Ukkola-Vuoti et al., 2013).

The microstructural underpinnings of the observed volumetric differences between brains of musicians and controls are an open matter of discussion (Draganski and May, 2008). Possible mechanisms include hippocampal cell proliferation, angiogenesis, microglia activation, axonal branching, myelin formation and synaptogenesis (Amunts et al., 1996; Zatorre et al., 2012). It will be essential for future neuroscientific research and potential therapeutical interventions to elucidate these mechanisms in further studies.

## Conclusions

In the current work, we examined the impact of long-term musical practice on measures of motor performance and on brain structure at a middle age. Professional musicians showed faster and better symmetric motor performances than controls, as well as a higher capability to control hand independence. Volumetric differences in brain structure between keyboard players and controls appeared in the sensorimotor cortices, the posterior half of the corpus callosum, the entorhinal cortex, and the superior parietal lobe, supposing a plastic effect through musician-specific requirements such as sight-playing, memorizing of scores and motor training. Importantly, the structural differences did not only appear between musicians and controls but also between two differently specialized and educated musician groups. Highly trained keyboard players with intensive practice regimes showed larger brain volumes than less specialized musicians, particularly in the sensorimotor cortices and corticospinal tract. Hence, intensity-dependent changes in brain structure were revealed. This allows the assumption of a measurable, training-related plasticity throughout the lifespan.

### Conflict of interest statement

The authors declare that the research was conducted in the absence of any commercial or financial relationships that could be construed as a potential conflict of interest.

## Footnotes

^1^“Music expresses that which cannot be said and on which it is impossible to be silent.” (Victor Hugo, “William Shakespeare,” Essay, 1864).

## References

[B1] Amunts K. Schlaug G. Jäncke L. Steinmetz H. Schleicher A. Dabringhaus A. (1997). Motor cortex and hand motor skills: structural compliance in the human brain. Hum. Brain Mapp. 5, 206–215 10.1002/(SICI)1097-0193(1997)5:3<206::AID-HBMS>3.0.CO;2-7 20408216

[B2] Amunts K. Schlaug G. Schleicher A. Steinmetz H. Dabringhaus A. Roland P. E. (1996). Asymmetry in the human motor cortex and handedness. Neuroimage 4, 216–222 10.1006/nimg.1996.0073 9345512

[B3] Amunts K. Schleicher A. Zilles K. (2007). Cytoarchitecture of the cerebral cortex - more than localization. Neuroimage 37, 1061–1065 10.1016/j.neuroimage.2007.02.037 17870622

[B4] Ashburner J. Friston K. J. (2005). Unified segmentation. Neuroimage 26, 839–851 10.1016/j.neuroimage.2005.02.018 15955494

[B5] Bangert M. Schlaug G. (2006). Specialization of the specialized in features of external human brain morphology. Eur. J. Neurosci. 24, 1832–1834 10.1111/j.1460-9568.2006.05031.x 17004946

[B6] Bartels C. Mertens N. Hofer S. Merboldt K. D. Dietrich J. Frahm J. (2008). Callosal dysfunction in amyotrophic lateral sclerosis correlates with diffusion tensor imaging of the central motor system. Neuromuscul. Disord. 18, 398–407 10.1016/j.nmd.2008.02.005 18456495

[B7] Bengtsson S. L. Nagy Z. Skare S. Forsman L. Forssberg H. Ullen F. (2005). Extensive piano practicing has regionally specific effects on white matter development. Nat. Neurosci. 8, 1148–1150 10.1038/nn1516 16116456

[B8] Bermudez P. Lerch J. P. Evans A. C. Zatorre R. J. (2009). Neuroanatomical correlates of musicianship as revealed by cortical thickness and voxel-based morphometry. Cereb. Cortex 19, 1583–1596 10.1093/cercor/bhn196 19073623

[B9] Bermudez P. Zatorre R. J. (2005). Differences in gray matter between musicians and nonmusicians. Ann. N.Y. Acad. Sci. 1060, 395–399 10.1196/annals.1360.057 16597791

[B10] Bürgel U. Amunts K. Hömke L. Mohlberg H. Gilsbach J. M. Zilles K. (2006). White matter fiber tracts of the human brain: three-dimensional mapping at microscopic resolution, topography and intersubject variability. Neuroimage 29, 1092–1105 10.1016/j.neuroimage.2005.08.040 16236527

[B11] Caspers S. Eickhoff S. Geyer S. Scheperjans F. Mohlberg H. Zilles K. (2008). The human inferior parietal lobule in stereotaxic space. Brain Struct. Funct. 212, 481–495 10.1007/s00429-008-0195-z 18651173

[B12] Caspers S. Geyer S. Schleicher A. Mohlberg H. Amunts K. Zilles K. (2006). The human inferior parietal cortex: cytoarchitectonic parcellation and interindividual variability. Neuroimage 33, 430–448 10.1016/j.neuroimage.2006.06.054 16949304

[B13] Chanda M. L. Levitin D. J. (2013). The neurochemistry of music. Trends Cogn. Sci. 17, 179–193 10.1016/j.tics.2013.02.007 23541122

[B14] Costa-Giomi E. (1999). The effects of three years of piano instruction on children's cognitive development. J. Res. Music Educ. 47, 198–212 10.2307/3345779

[B15] Dell'Acqua F. Catani M. (2012). Structural human brain networks: hot topics in diffusion tractography. Curr. Opin. Neurol. 25, 375–383 22766720 10.1097/WCO.0b013e328355d544

[B16] Draganski B. May A. (2008). Training-induced structural changes in the adult human brain. Behav. Brain Res. 192, 137–142 10.1016/j.bbr.2008.02.015 18378330

[B17] Elbert T. Pantev C. Wienbruch C. Rockstroh B. Taub E. (1995). Increased cortical representation of the fingers of the left hand in string players. Science 270, 305–307 10.1126/science.270.5234.305 7569982

[B18] Eriksson P. S. Perfilieva E. Bjork-Eriksson T. Alborn A. M. Nordborg C. Peterson D. A. (1998). Neurogenesis in the adult human hippocampus. Nat. Med. 4, 1313–1317 10.1038/3305 9809557

[B19] Evans A. C. Janke A. L. Collins D. L. Baillet S. (2012). Brain templates and atlases. Neuroimage 62, 911–922 10.1016/j.neuroimage.2012.01.024 22248580

[B20] Fyhn M. Molden S. Witter M. P. Moser E. I. Moser M. B. (2004). Spatial representation in the entorhinal cortex. Science 305, 1258–1264 10.1126/science.1099901 15333832

[B21] Gaser C. Schlaug G. (2003). Brain structures differ between musicians and non-musicians. J. Neurosci. 23, 9240–9245 14534258 10.1523/JNEUROSCI.23-27-09240.2003 PMC6740845

[B22] Geyer S. (2003). The Microstructural Border Between the Motor and the Cognitive Domain in the Human Cerebral Cortex. Wien: Springer 10.1007/978-3-642-18910-4 14750415

[B23] Geyer S. Ledberg A. Schleicher A. Kinomura S. Schormann T. Burgel U. (1996). Two different areas within the primary motor cortex of man. Nature 382, 805–807 10.1038/382805a0 8752272

[B24] Geyer S. Schleicher A. Zilles K. (1999). Areas 3a, 3b, and 1 of human primary somatosensory cortex: 1. Microstructural organization and interindividual variability. Neuroimage 10, 63–83 10.1006/nimg.1999.0440 10385582

[B25] Geyer S. Schormann T. Mohlberg H. Zilles K. (2000). Areas 3a, 3b, and 1 of human primary somatosensory cortex: 2. Spatial normalization to standard anatomical space. Neuroimage 11, 684–696 10.1006/nimg.2000.0548 10860796

[B26] Goebl W. Palmer C. (2008). Tactile feedback and timing accuracy in piano performance. Exp. Brain Res. 186, 471–479 10.1007/s00221-007-1252-1 18193412

[B27] Granert O. Peller M. Jabusch H. C. Altenmüller E. Siebner H. R. (2011). Sensorimotor skills and focal dystonia are linked to putaminal grey-matter volume in pianists. J. Neurol. Neurosurg. Psychiatry 82, 1225–1231 10.1136/jnnp.2011.245811 21705464

[B28] Grefkes C. Geyer S. Schormann T. Roland P. Zilles K. (2001). Human somatosensory area 2: observer-independent cytoarchitectonic mapping, interindividual variability, and population map. Neuroimage 14, 617–631 10.1006/nimg.2001.0858 11506535

[B29] Groussard M. La Joie R. Rauchs G. Landeau B. Chételat G. Viader F. (2010). When music and long-term memory interact: effects of musical expertise on functional and structural plasticity in the hippocampus. PLoS ONE 5:e13225 10.1371/journal.pone.0013225 20957158 PMC2950159

[B30] Han Y. Yang H. Lv Y. T. Zhu C. Z. He Y. Tang H. H. (2009). Gray matter density and white matter integrity in pianists brain: a combined structural and diffusion tensor MRI study. Neurosci. Lett. 459, 3–6 10.1016/j.neulet.2008.07.056 18672026

[B31] Herholz S. C. Zatorre R. (2012). Musical training as a framework for brain plasticity: behavior, function, and structure. Neuron 76, 486–502 10.1016/j.neuron.2012.10.011 23141061

[B32] Hetland L. (2000). Learning to make music enhances spatial reasoning. J. Aesthet. Educ. 34, 179–238 10.2307/3333643

[B33] Hofer S. Frahm J. (2006). Topography of the human corpus callosum revisited - comprehensive fiber tractography using diffusion tensor magnetic resonance imaging. Neuroimage 32, 989–994 10.1016/j.neuroimage.2006.05.044 16854598

[B34] Holmes C. J. Hoge R. Collins L. Woods R. Toga A. W. Evans A. C. (1998). Enhancement of MR images using registration for signal averaging. J. Comp. Assist. Tomogr. 22, 324–333 10.1097/00004728-199803000-00032 9530404

[B35] Hömke L. (2006). A multigrid method for anisotropic PDEs in elastic image registration. Numer. Linear Algebra Appl. 13, 215–229 10.1002/nla.477

[B36] Hutchinson S. Lee L. H. L. Gaab N. Schlaug G. (2003). Cerebellar volume of musicians. Cereb. Cortex 13, 943–949 10.1093/cercor/13.9.943 12902393

[B37] Hyde K. L. Lerch J. Norton A. Forgeard M. Winner E. Evans A. C. (2009a). Musical training shapes structural brain development. J. Neurosci. 29, 3019–3025 10.1523/JNEUROSCI.5118-08.2009 19279238 PMC2996392

[B38] Hyde K. L. Lerch J. Norton A. Forgeard M. Winner E. Evans A. C. (2009b). The effects of musical training on structural brain development. Ann. N.Y. Acad. Sci. 1169, 182–186 10.1111/j.1749-6632.2009.04852.x 19673777

[B39] Imfeld A. Oechslin M. S. Meyer M. Loenneker T. Jäncke L. (2009). White matter plasticity in the corticospinal tract of musicians: a diffusion tensor imaging study. Neuroimage 46, 600–607 10.1016/j.neuroimage.2009.02.025 19264144

[B40] Jabusch H. C. Alpers H. Kopiez R. Vauth H. Altenmüller E. (2008). The influence of practice on the development of motor skills in pianists: a longitudinal study in a selected motor task. Hum. Mov. Sci. 28, 74–84 10.1016/j.humov.2008.08.001 18845349

[B41] Jäncke L. Schlaug G. Steinmetz H. (1997). Hand skill asymmetry in professional musicians. Brain Cogn. 34, 424–432 10.1006/brcg.1997.0922 9292190

[B42] Jenkinson M. Bannister P. Brady M. Smith S. (2002). Improved optimization for the robust and accurate linear registration and motion correction of brain images. Neuroimage 17, 825–841 10.1006/nimg.2002.1132 12377157

[B43] Jones D. K. Knösche T. R. Turner R. (2012). White matter intergrity, fiber count, and other fallacies: the do's and don'ts of diffusion MRI. Neuroimage 73, 239–254 10.1016/j.neuroimage.2012.06.081 22846632

[B44] Kalbe E. Kessler J. Calabrese P. Smith R. Passmore A. P. Brand M. (2004). DemTect: a new, sensitive cognitive screening test to support the diagnosis of mild cognitive impairment and elderly dementia. Int. J. Geriatr. Psychiatry 19, 136–143 10.1002/gps.1042 14758579

[B45] Karni A. Meyer G. Jezzard P. Adams M. M. Turner R. Ungerleider L. G. (1995). Functional MRI evidence for adult motor cortex plasticity during motor skill learning. Nature 377, 155–158 10.1038/377155a0 7675082

[B46] Keenan J. P. Thangaraj V. Halpern A. R. Schlaug G. (2001). Absolute pitch and planum temporale. Neuroimage 14, 1402–1408 10.1006/nimg.2001.0925 11707095

[B47] Kraus N. Chandrasekaran B. (2010). Music training for the development of auditory skills. Nat. Rev. Neurosci. 11, 599–605 10.1038/nrn2882 20648064

[B48] Lavenex P. Banta Lavenex P. (2013). Building hippocampal circuits to learn and remember: insights into the development of human memory. Behav. Brain. Res. 254, 8–21 10.1016/j.bbr.2013.02.007 23428745

[B49] Lee D. J. Chen Y. Schlaug G. (2003). Corpus callosum: musician and gender effects. Neuroreport 14, 205–209 10.1097/00001756-200302100-00009 12598730

[B50] Li S. Han Y. Wang D. Yang H. Fan Y. Lv Y. (2010). Mapping surface variability of the central sulcus in musicians. Cereb. Cortex 20, 25–33 10.1093/cercor/bhp074 19433652

[B51] Luders E. Gaser C. Jancke L. Schlaug G. (2004). A voxel-based approach to gray matter asymmetries. Neuroimage 22, 656–664 10.1016/j.neuroimage.2004.01.032 15193594

[B52] Lv Y. T. Yang H. Wang D. Y. Li S. Y. Han Y. Zhu C. Z. (2008). Correlations in spontaneous activity and gray matter density between left and right sensorimotor areas of pianists. Neuroreport 19, 631–634 10.1097/WNR.0b013e3282fa6da0 18382276

[B53] Maguire E. A. Gadian D. G. Johnsrude I. S. Good C. D. Ashburner J. Frackowiak R. S. J. (2000). Navigation-related structural change in the hippocampi of taxi drivers. Proc. Natl. Acad. Sci.U.S.A. 97, 4398–4403 10.1073/pnas.070039597 10716738 PMC18253

[B54] Mathalon D. H. Sullivan E. V. Rawles J. M. Pfefferbaum A. (1993). Correction for head size in brain-imaging measurements. Psychiatry Res. 50, 121–139 10.1016/0925-4927(93)90016-B 8378488

[B55] Morley A. P. Narayanan M. Mines R. Molokhia A. Baxter S. Craig G. (2012). AVPR1A and SLC*6A4* polymorphisms in choral singers and non-musicians: a gene association study. PLoS ONE 7:e31763 10.1371/journal.pone.0031763 22384070 PMC3285181

[B56] Münte T. F. Altenmüller E. Jäncke L. (2002). The musician's brain as a model of neuroplasticity. Nat. Rev. Neurosci. 3, 473–478 12042882 10.1038/nrn843

[B57] Oldfield R. C. (1971). The assessment and analysis of handedness: the Edinburgh inventory. Neuropsychologia 9, 97–113 10.1016/0028-3932(71)90067-4 5146491

[B58] Öztürk H. Tascioglu B. Aktekin M. Kurtoglu Z. Erden I. (2002). Morphometric comparison of the human corpus callosum in professional musicians and non-musicians by using *in vivo* magnetic resonance imaging. J. Neuroradiol. 29, 29–34 11984475

[B59] Park H. Lee S. Kim H. J. Ju Y. S. Shin J. Y. Hong D. (2012). Comprehensive genomic analyses associate UGT8 variants with musical ability in a Mongolian population. J. Med. Gen. 49, 747–752 10.1136/jmedgenet-2012-101209 23118445 PMC3512346

[B60] Pascual-Leone A. Nguyet D. Cohen L. G. Brasil-Neto J. P. Cammarota A. Hallett M. (1995). Modulation of muscle responses evoked by transcranial magnetic stimulation during the acquisition of new fine motor skills. J. Neurophysiol. 74, 1037–1045 7500130 10.1152/jn.1995.74.3.1037

[B61] Pau S. Jahn G. Sakreida K. Domin M. Lotze M. (2013). Encoding and recall of finger sequences in experienced pianists compared with musically naive controls: A combined behavioral and functional imaging study. Neuroimage 64, 379–387 10.1016/j.neuroimage.2012.09.012 22982586

[B62] Penhune V. B. Watanabe D. Savion-Lemieux T. (2005). The effect of early musical training on adult motor performance. Ann. N.Y. Acad. Sci. 1060, 265–268 10.1196/annals.1360.049 16597774

[B63] Pieperhoff P. Südmeyer M. Hömke L. Zilles K. Schnitzler A. Amunts K. (2008). Detection of structural changes of the human brain in longitudinally acquired MR images by deformation field morphometry: methodological analysis, validation and application. Neuroimage 43, 269–287 10.1016/j.neuroimage.2008.07.031 18706506

[B64] Rencher A. C. (2002). Methods of Multivariate Analysis. New Jersey, NJ: John Wiley and Sons 10.1002/0471271357

[B65] Rencher A. C. Scott D. T. (1990) Assessing the contribution of individual variables following the rejection of a multivariate hypothesis. Commun. Stat. Simul. Comput. 19, 535–553 10.1080/03610919008812874

[B66] Ridding M. C. Brouwer B. Nordstrom M. A. (2000). Reduced interhemispheric inhibition in musicians. Exp. Brain. Res. 133, 249–253 10.1007/s002210000428 10968226

[B67] Rosenkranz K. Williamon A. Rothwell J. C. (2007). Motorcortical excitability and synaptic plasticity is enhanced in professional musicians. J. Neurosci. 27, 5200–5206 10.1523/JNEUROSCI.0836-07.2007 17494706 PMC6672373

[B68] Salimpoor V. N. van den Bosch I. Kovacevic N. McIntosh A. R. Dagher A. Zatorre R. J. (2013). Interactions between the nucleus accumbens and auditory cortices predict music reward value. Science 340, 216–219 10.1126/science.1231059 23580531

[B69] Scheperjans F. Hermann K. Eickhoff S. B. Amunts K. Schleicher A. Zilles K. (2008a). Observer-independent cytoarchitectonic mapping of the human superior parietal cortex. Cereb. Cortex 18, 846–867 10.1093/cercor/bhm116 17644831

[B70] Scheperjans F. Eickhoff S. B. Hömke L. Mohlberg H. Hermann K. Amunts K. (2008b). Probabilistic maps, morphometry, and variability of cytoarchitectonic areas in the human superior parietal cortex. Cereb. Cortex 18, 2141–2157 10.1093/cercor/bhm241 18245042 PMC3140197

[B71] Schlaug G. Jäncke L. Huang Y. Staiger J. F. Steinmetz H. (1995). Increased corpus callosum size in musicians. Neuropsychologia 33, 1047–1055 10.1016/0028-3932(95)00045-5 8524453

[B72] Schlaug G. Norton A. Overy K. Winner E. (2005). Effects of music training on the child's brain and cognitive development. Ann. N.Y. Acad. Sci. 1060, 219–230 10.1196/annals.1360.015 16597769

[B73] Schmithorst V. J. Wilke M. (2002). Differences in white matter architecture between musicians and non-musicians: a diffusion tensor imaging study. Neurosci. Lett. 321, 57–60 10.1016/S0304-3940(02)00054-X 11872256

[B74] Schneider P. Sluming V. Roberts N. Bleeck S. Rupp A. (2005). Structural, functional, and perceptual differences in Heschl's gyrus and musical instrument preference. Ann. N.Y. Acad. Sci. 1060, 387–394 10.1196/annals.1360.033 16597790

[B75] Sergent J. (1993). Mapping the musician brain. Hum. Brain Mapp. 1, 20–38 10.1002/hbm.460010104

[B77] Sled J. G. Zijdenbos A. P. Evans A. C. (1998). A nonparametric method for automatic correction of intensity nonuniformity in MRI data. IEEE Trans. Med. Imaging 17, 87–97 10.1109/42.668698 9617910

[B78] Sluming V. Barrick T. Howard M. Cezayirli E. Mayes A. Roberts N. (2002). Voxel-based morphometry reveals increased gray matter density in Broca's area in male symphony orchestra musicians. Neuroimage 17, 1613–1622 10.1006/nimg.2002.1288 12414299

[B79] Steele C. Bailey J. A. Zatorre R. J. Penhune V. B. (2013). Early musical training and white-matter plasticity in the corpus callosum: evidence for a sensitive period. J. Neurosci. 33, 1282–1290 10.1523/JNEUROSCI.3578-12.2013 23325263 PMC6704889

[B80] Steingrüber H. J. (1971). Zur Messung der Händigkeit. Z. Exp. Angew. Physiol. 18, 337–357 5561973

[B81] Stewart L. (2008). Do musicians have different brains? Clin. Med. 8, 304–308 10.7861/clinmedicine.8-3-304 18624043 PMC4953838

[B82] Stewart L. Henson R. Kampe K. Walsh V. Turner R. Frith U. (2003). Brain changes after learning to read and play music. Neuroimage 20, 71–83 10.1016/S1053-8119(03)00248-9 14527571

[B83] Suthana N. Haneef Z. Stern J. Mukamel R. Behnke E. Knowlton B. (2012). Memory enhancement and deep-brain stimulation of the entorhinal area. N. Engl. J. Med. 366, 502–510 10.1056/NEJMoa1107212 22316444 PMC3447081

[B84] Theusch E. Gitschier J. (2011). Absolute pitch twin study and segregation analysis. Twin Res. Hum. Genet. 14, 173–178 10.1375/twin.14.2.173 21425900

[B85] Udtaisuk D. B. (2005). A Theoretical Model of Piano Sightplaying Com-ponents. Dissertation University of Missouri-Columbia.

[B86] Ukkola-Vuoti L. Kanduri C. Oikkonen J. Buck G. Blancher C. Raijas P. (2013). Genome-wide copy number variation analysis in extended families and unrelated indiciduals characterized for musical aptitude and creaticity in music. PLoS ONE 8:e56356 10.1371/journal.pone.0056356 23460800 PMC3584088

[B87] Vaina L. M. Solomon J. Chowdhury S. Sinha P. Belliveau J. W. (2001). Functional neuroanatomy of biological motion perception in humans. Proc. Natl. Acad. Sci. U.S.A. 98, 11656–11661 10.1073/pnas.191374198 11553776 PMC58785

[B88] Wahl M. Lauterbach-Soon B. Hattingen E. Jung P. Singer O. Volz S. (2007). Human motor corpus callosum: topography, somatotopy, and link between microstructure and function. J. Neurosci. 27, 12132–12138 10.1523/JNEUROSCI.2320-07.2007 17989279 PMC6673264

[B89] Witelson S. F. (1989). Hand and sex differences in the isthmus and genu of the genu of the human corpus callosum: a postmortem morphological study. Brain 112, 799–835 10.1093/brain/112.3.799 2731030

[B90] Wolpert D. M. Goodbody S. J. Husain M. (1998). Maintaining internal representations: the role of the human superior parietal lobe. Nat. Neurosci. 1, 529–533 10.1038/2245 10196553

[B91] Yousry T. A. Schmid U. D. Alkadhi H. Schmidt D. Peraud A. Buettner A. (1997). Localization of the motor hand area to a knob on the precentral gyrus. A new landmark. Brain 120, 141–157 10.1093/brain/120.1.141 9055804

[B92] Zatorre R. J. Fields R. D. Johansen-Berg H. (2012). Plasticity in gray and white: neuroimaging changes in brain structure during learning. Nat. Neurosci. 15, 528–536 10.1038/nn.3045 22426254 PMC3660656

[B93] Zendel B. R. Alain C. (2013). The influence of lifelong musicianship on neurophysiological measures of concurrent sound segregation. J. Cogn. Neurosci. 25, 503–516 10.1162/jocn_a_00329 23163409

[B94] Zilles K. Amunts K. (2010). Centenary of Brodmann's map - conception and fate. Nat. Rev. Neurosci. 22, 139–145 10.1038/nrn2776 20046193

